# Potential benefits of a virtual, home-based combined exercise and mindfulness training program for HSC transplant survivors: a single-arm pilot study

**DOI:** 10.1186/s13102-022-00554-7

**Published:** 2022-09-05

**Authors:** David D. F. Ma, Kate Fennessy, David Kliman

**Affiliations:** 1 Department of Haematology and Bone Marrow Transplant, St Vincent’s Hospital Sydney, 390 Victoria Street, Darlinghurst, NSW Australia; 2 Present Address: Department of Haematology, Royal North Shore Hospital, Sydney, NSW Australia

**Keywords:** Allogeneic HCT, Physical exercise, Mindfulness, Telehealth, QOL

## Abstract

**Purpose:**

Impaired quality of life (QOL) including reduced physical fitness is a recognized late effect of hemopoietic cell transplantation (HCT). Guided exercise and mindfulness-based stress management (MBSM) programs have shown promise, mainly in the inpatient setting. We aimed to examine the feasibility of a virtual, home-based, combined exercise and MBSM program.

**Methods:**

Patients attending post-HCT clinic were invited to participate in this single-arm pre-post study. Eligibility criteria included age 18–75 years, > 6 months post allogeneic HCT. Consented participants attended an in-person session, followed by weekly exercise and MBSM training for 6 weeks via videoconferencing. Assessments were performed pre-training, and at 3-, 6- and 12-months and compared using a linear mixed effects model.

**Results:**

21 of 24 patients consenting to the study completed the program (median age 56 years [IQR 46–62], median time post-HCT 37 months [IQR 26–46]). Six-minute walk test scores were significantly higher at 3 (mean difference 79.6, 95%CI 28–131, ES 0.55) and 12 months (mean difference 48.4, 95%CI 13–84, ES 0.33) compared to baseline. Sit-to-stand test was significantly higher at 3 (mean difference 4.4, 95%CI 1.4–7.4, ES 0.68) and 12 months (mean difference 3.9, 95%CI 0.24–7.6, ES 0.61). Dominant hand grip was significantly stronger at 3 (mean difference 0.16, 95%CI 0.04–0.28, ES 0.45), and 12 months (mean difference 0.21, 95%CI 0.08–0.24, ES 0.62). Significantly higher FACT-BMT total (mean difference 6.9, 95%CI 1.5–12.4, ES 0.49) and FACT-G scores (mean difference 5.2, 95%CI 1.4–9.1, ES 0.48) were found at 3 months. Over 80% of participants rated the virtual combined modal program highly and no adverse events were reported.

**Conclusion:**

A 6-week virtual, home-based exercise and MBSM program was an acceptable, and potentially effective intervention for sustained improvement of some physical capacity and QOL outcomes in HCT survivors. Virtual-based healthcare service is highly relevant particularly during pandemics. To our knowledge, this study has the longest follow-up observation period for Internet based combined modality training program reported to date and warrants additional investigation.

*Trial Registration* Research protocol approved by St Vincent’s Hospital Ethics Committee (HREC 12/SVH/175), approved 27/09/2012, trial commenced 24/05/13 and the first participant 07/06/13. Retrospectively registered with ANZCTR (ACTRN12613001054707) 23/09/2013.

**Supplementary Information:**

The online version contains supplementary material available at 10.1186/s13102-022-00554-7.

## Introduction

Allogeneic haematopoietic stem cell transplantation (HSCT) is an effective treatment for many patients with haematological cancers and non-malignant diseases. The life expectancy of transplant patients now approaches that of the matched non-transplant population [1, 2]. Many patients who survive over 5 years post-transplant report a high quality of life (QoL) and functional state [3]. However, functional deficits, emotional distress and life-threatening health conditions such as cardiovascular disease, diabetes and cancer are reported at a greater prevalence in this population than in age-matched peers, or in survivors of other cancer treatments [4–8]. This highlights the need for ongoing healthcare for cancer and HSCT survivors, with potential benefits to patients, families, the workforce, and the wider community [9].

Preventative non-pharmaceutical interventions show promise to improve the physical capacity, QoL and functioning of HSCT patients. Supervised exercise programs have demonstrated physical and psychological benefits for haematological cancer patients undergoing transplant [10–14], with improved aerobic fitness, subjective physical well-being, fatigue and distress in patients both pre-HSCT [10], or within 6 months post HSCT [15, 16] and which may be sustained for up to 6 months post intervention [15]. Indicators of health benefits, such as fewer days of hospitalisation, were reported for allogeneic transplant recipients receiving myeloablative conditioning [17, 18]. Mindfulness-Based Stress Management (MBSM) interventions are increasingly shown to be effective in improving pain, nausea, mood disturbance, fatigue, and anxiety for adult cancer patients, and increase positive affect in patients in the early post-transplant period (< 6 months) [19–22]. However, information on the efficacy of MBSM interventions in longer-term post-HSCT follow-up is sparse.

It has been postulated that the benefits of combined physical exercise (PE) and MBSM programs may be synergistic: stress management may facilitate willingness to engage in exercise, with behavioural change in turn supported by an increased sense of self-efficacy resulting from achievement of exercise goals. Combined modality (PE + MBSM) intervention has been trialled in early post-transplant patients but thus far has most have not demonstrated benefit with the combined approach [23, 24]. The one positive study only assessed responses within 12 weeks of the intervention, with no long term follow up to evaluate sustained improvements [25]. The self-guided nature, timing and duration of the programs conducted in HSCT patients may have contributed to these non-significant findings. Providing a supervised, individualised intervention over an extended period of time, and further removed from the acute transplant phase may be able to overcome the limitations of previous studies.

HSCT is mainly performed in a few specialist centres in capital cities, with significant barriers to accessibility for patients living in remote areas. Approximately 45% of Australian HSCT patients live > 50 km from their treatment centre, a factor associated with lower survival rates 1 year post allo-HSCT [26]. Attendance at a long-term post-transplant follow-up clinic by these patients counters the adverse effect of distance [27]. However, frequent follow-up visits may create additional burden for patients and carers, and there is limited specialised support for non-metropolitan patients between visits.

Telehealth capabilities can be used to overcome some of these deficiencies in meeting patient needs. In Australia, videoconferencing has been used for medical consultations and interventions [28, 29], and effectively delivers psycho-educational programs to HSCT survivors [30–32]. The use of this technology in healthcare has been accelerated during the current COVID-19 pandemic, including in the cancer survivor population, given the potential to reduce virus exposure and transmission [33, 34]. However, the efficacy and cost-effectiveness of telehealth interventions to meet the needs of patients residing in the community remains to be rigorously evaluated [35].

The current study focused on patients > 6 months post-HSCT, a patient population with different challenges in implementing intervention and lifestyle changes compared to hospital in-patients. The aim of this single centre pilot study is to evaluate the feasibility and benefits of a 6-week personalised combined PE and MBSM telehealth intervention in this group of patients on physical fitness, psychological wellbeing, and adherence at 3-, 6- and 12-months post-intervention.

## Materials/subjects and methods

### Study design and population

**Fig. 1:**
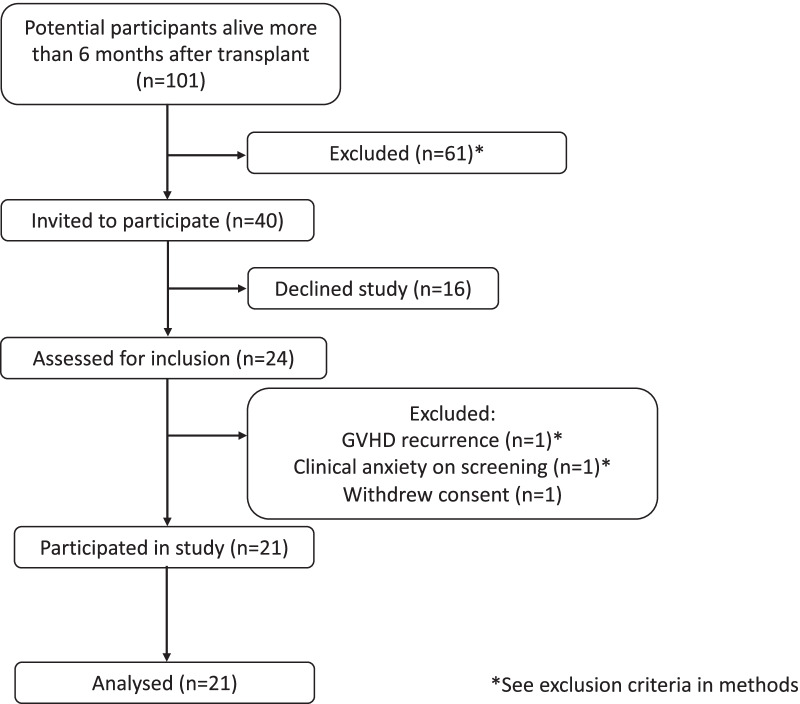
Participant CONSORT diagram depicting flow for recruitment and adherence. * see exclusion criteria in Methods section

We designed a single centre, single arm pre-post study to assess the feasibility of the planned combined intervention. Patients of St Vincent’s Hospital Sydney, Australia, who were > 6 months post allogeneic (allo) HSCT and aged over 18 years, with the ability to complete the patient- reported outcome questionnaires and physical assessments were invited to participate by phone between May and July 2013 (Fig. 1). Participation was approved by the patient’s primary specialist prior to screening. Patients with severe graft-versus-host disease (GVHD), significant cardiac disease, disease relapse, or other medical issues requiring regular clinic visits were excluded. Potential participants were screened using the Hospital Anxiety and Depression Subscale (HADS) [36] and those with a score of ≥ 15 (severe) were excluded and referred for psychological treatment. Patients with a life expectancy of < 6 months, or insufficient basic skill to use a computer for audio-visual conferencing via the internet were excluded. The research protocol was approved by the St Vincent’s Hospital Ethics Committee (HREC 12/SVH/175, 27/09/2012) and conducted in accordance with the Declaration of Helsinki. The trial was retrospectively registered 23/09/2013 with the Australian and New Zealand Clinical Trials Registry (ACTRN12613001054707,).

### Intervention

Consented participants attended a half-day in-person screening and introductory session at the transplant clinic, which included completion of baseline assessments, familiarisation with videoconferencing technology, questions related to health and safety, and scheduling of future training and assessment sessions. Each patient participated in a one-to-one session with the trainers (rehabilitation physiotherapist and clinical psychologist) to complete initial assessments, education, goal setting and training in MBSM and PE techniques. All measurements were trainer-administered at baseline and at the time of each subsequent assessment. Participants were provided with 5 voice-recordings of guided mindfulness tracks for skill practice on a USB drive, and a hand grip dynamometer and resistance bands for home exercise practice. A hard-copy booklet was supplied which included information about stress management skills and exercise, as well as goals, a practice log and follow-up web appointment schedule to be completed.

The initial in-person training session was followed by five one-on-one personalised training sessions for each of the PE and MBSM components of the program. These sessions were conducted via videoconference using Skype at pre-scheduled times on a weekly basis and, each was approximately 60 min in duration. The separate PE and MBSM sessions were delivered consecutively or on different days of the week according to participant preference. Details of the interventions are included in the Additional files 1, 2, & 3.

The PE training sessions included modelling and coaching physical exercises, reviewing progress, and goal setting. Training was comprised of moderate intensity aerobic and resistance exercises with stretching, adapted to the participants’ initial fitness level and gradually increased in intensity and duration, or reduced and adapted according to patient needs and ability. The goal of cardio-vascular exercise was 25–30 min per session, 3–5 times per week and could include walking, running, swimming or cycling. Resistance exercises included 2–3 sets of 8–12 repetitions with resistance bands (See Additional file 1: appendix A–E). The target during cardiovascular exercise and resistance/strength training was to reach ratings between 11 and 14 of the Borg Rating of Perceived Exertion (RPE) scale [37]. Participants were to report any adverse events and given recommendations for regular practice during and at the end of the supervised training period.

The MBSM sessions focused on stress management, psycho-education about stress, anxiety and low mood symptoms, individual cognitive and behavioural coping strategies and the practice of skills to improve well-being. Participants were trained in grounding and mindfulness strategies, following a standard MBSM methodology [19, 38]. Each session consisted of guided experiential mindfulness exercises (e.g., focus on the breath, body scan, sounds), psychoeducation (e.g., on topics such as coping strategies or problem solving) and review of homework exercises. The program aimed to, firstly, increase present moment awareness and recognize entanglement with one’s thoughts and emotions; and secondly, to teach mindfulness and acceptance as an alternative strategy for dealing with difficult thoughts and feelings, and how these may be used to facilitate value-based actions. Participants were asked about any adverse events, such as injuries, and provided with recommendations for regular practice during and at the end of the supervised training period. For both exercise and MBSR components, self-reported practice was recorded in hard copy participant files and barriers to practice were addressed via structured problem-solving techniques. Attendance at both the PE and MBSM Skype sessions were logged for assessing adherence.

### Outcome measures

Measures were collected at baseline (T1), post 6-week training (T2), and at 3- (T3), 6- (T4) and 12- (T5) months post-training. Objective assessments of physical performance included: 6-Minute Walk Test (6-MWT) [39], the Bruce Test [40] Hand Grip Strength Measures [41] and the 30 s Sit-to-Stand Test (STS) [42]. The measurements were recorded by the assessor via a videoconferencing application, Skype, with the participant remaining at their home. Further details of the interventions and assessments are included in the Additional files 1, 2, & 3.

Self-report measures of functioning and quality of life included: the Hospital Anxiety and Depression Score (HADS), Godin-Shepard Leisure Time Questionnaire [43]. Pittsburgh Sleep Quality Index [44] and the Functional Assessment of Cancer Therapy- Bone Marrow Transplant (FACT-BMT) [45]. At 6-months post-training, participants were requested to provide their evaluation of the 6-week training program via an 8 item questionnaire with 6-point Likert scales, and free response (see Additional file 4: Fig. S5). A mean score of 3.5 or above was considered positive as these responses were denoted by positively valanced language, eg, “3 = many useful elements, 4 = very much and 5 = a great deal”. We determined the intervention to be feasible if > 50% of eligible patients enrolled in the study and > 50% of participants completed the baseline assessments as well as completed 5/6 weeks of the multimodal intervention sessions, consistent with metrics used in feasibility trials of exercise and behavioral intervention studies. We established the intervention to be acceptable if mean participants’ ratings of ease and utility scores (Q5–Q7 of the participant feedback at 6 months post-intervention) were ≥ 3.5/5 on 6-point Likert scales (0 = not at all, 5 = a great deal).

### Statistical analysis

For comparison of outcome measurements between pre- and post- intervention time points, linear mixed-effects model was used. This maximum likelihood-based approach utilizes all acquired data points and manages missing data points by missing at random (MAR) assumption. Model checking was performed based on standardized residues. Logarithmic transformations were performed if after examining the residual analysis the heterogeneity of variance assumption was violated. *P* values were adjusted using Bonferroni method for multiple comparisons, with a score of < 0.05 considered significant. All analyses including the effect size (ES) were carried out using Social Science Statistics calculators (www.socscistatistics.com) and GraphPad Prism 8 (GraphPad Prism, La Jolla, CA, USA).

## Results

Of the 101 patients initially screened, 40 were eligible for inclusion. Twenty-four of the 40 (60%) consented to participate, with 21 (87.5%) completing the 6-week PE and MBSM training (Fig. 1). Adherence to training sessions (ratio of the number of training sessions performed relative to the number of prescribed sessions (*n* = 12) was 88.6%

Participants were a median 53 years of age at the time of recruitment (range 33–73). One third of the enrolled cohort resided rurally, defined as > 50 km away from our hospital. The median time since transplantation was 37 (range 13–68) months at time of enrolment. Twenty-nine percent had experienced Grade II-IV acute GVHD and 48% chronic GVHD (Table 1).

**Table 1:** Patient demographic data

Characteristic	Number (total = 21)
*Age at recruitment (years)*	
Median	56
Range (IQR)	33–73 (46–62)
*Time from transplant (months)*	
Median	37
Range (IQR)	13–68 (26–46)
*Sex*	
Male	12 (57%)
Female	9 (43%)
*Location*	
Urban	14 (67%)
Rural/remote	7 (33%)
*Disease indication*	
Acute myeloid leukemia	12 (57%)
Non-hodgkin lymphoma	5 (24%)
Other	4 (19%)
*Donor type*	
Sibling	9 (43%)
Unrelated	12 (57%)
*Conditioning intensity*	
Myeloablative	7 (33%)
*Reduced intensity*	14 (67%)
Acute GVHD (Grade II-IV)	
Yes	6 (29%)
No	15 (71%)
*Chronic GVHD*	
Yes	10 (48%)
No	11 (52%)

*Rural is defined as residing > 50 km away from our hospital

**Fig. 2:**
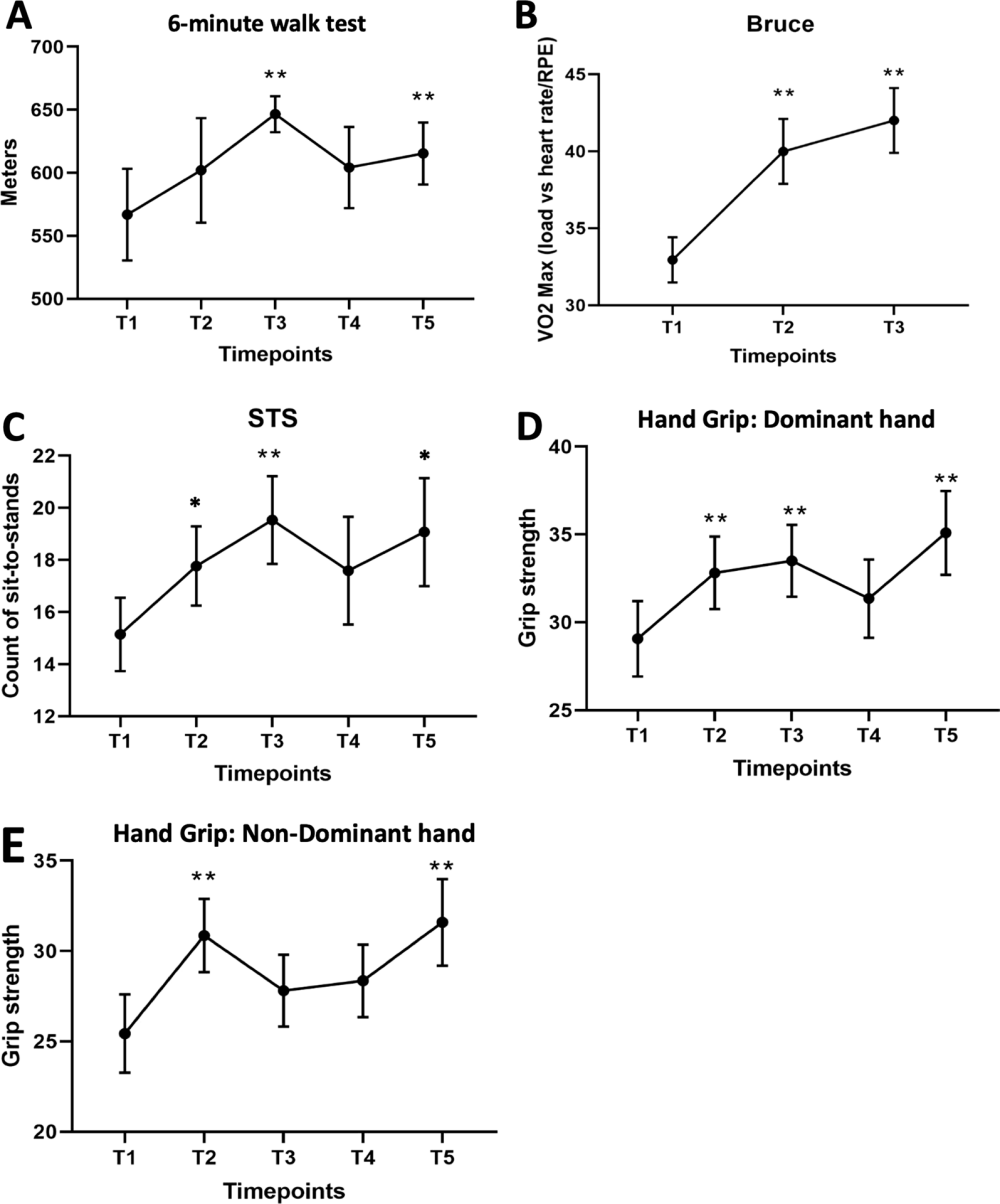
**a**, **b**: Participant improvement on cardiovascular measures, where higher scores indicate improved performance, **p < .01. **a** the 6-min walk test (6MWT), and **b** Bruce test. **c** Participant improvement on a lower-body strength test, the Sit to Stand (STS) test, where higher scores indicate improved strength, *p < .05, **p < .01. **d**, **e** Participant improvement on an upper-body strength test, the Hand Grip test, **p < .01. Performed for both **d** participant’s dominant hand, and **e** non-dominant hand

**Fig. 3:**
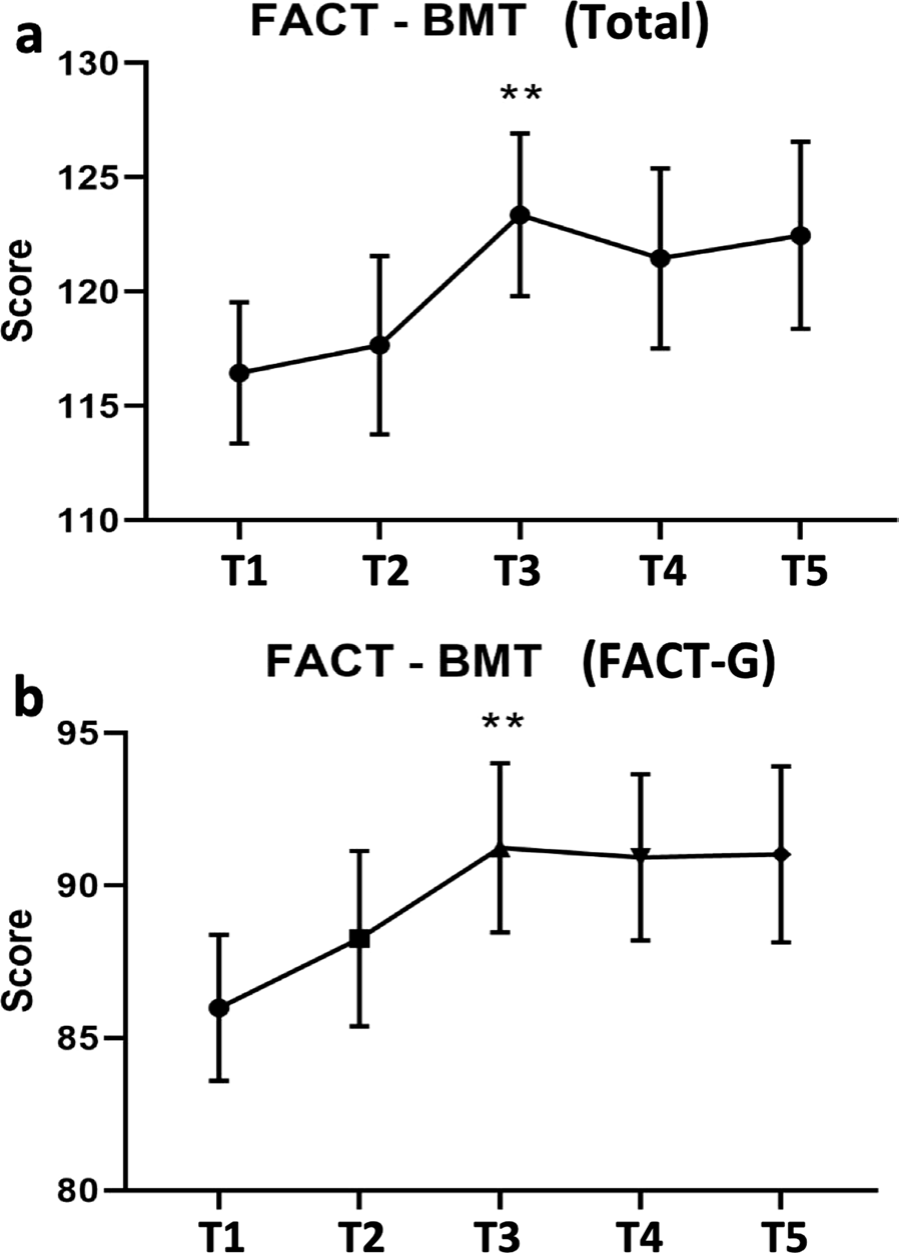
Participant improvement on the **a** FACT-BMT total and **b** FACT-G subscales measuring self-reported QOL. Higher scores indicate improvements in perceived QOL **p < .01

Participants’ physical fitness, level of symptoms of anxiety and depression, and QOL at 3 months (T3) post completion of the training were compared to baseline (T1) (Figs. 2, 3, and Table 2). Increased cardiovascular fitness was demonstrated in the 6-MWT and Bruce tests. The 6-MWT scores were significantly higher at T3 (M = 646.5, SD = 53.34, ES = 0.55) and T5 (M = 615.33, SD = 94.95, ES = 0.33) compared to T1 (M = 566.94, SD = 145.22; Fig. 2a). Similarly for the Bruce test, significant effects were observed at T2 (M = 39.99, SD = 7.88, ES = 1.0), and at T3 (M = 42.01, SD = 7.85, ES = 1.41; Fig. 2b) compared to T1 (M = 32.95, SD = 6.42). Bruce test was discontinued after T3 as it involved transplant centre visits by participants.

**Table 2:** Patient assessment data following intervention

Outcome measure	T1 mean (SD)	Mean 2 (SD)	Mean diff.	95.00% CI of diff.	Adjust *p* value	Effect size
*6-MWT*						
T1 vs. T2	566.9 (145.2)	602 (143.2)	− 35.06	− 84.75 to 14.63	0.237	0.242
T1 vs. T3		646.5 (55.3)	− 79.56	− 131.1 to − 28.01	0.004**	0.55
T1 vs. T4		604.3 (124.4)	− 37.33	− 111.5 to 36.80	0.628	0.26
T1 vs. T5		615.3 (94.9)	− 48.4	− 83.90 to − 12.89	0.009**	0.33
*Bruce*						
T1 vs. T2	32.95 (6.42)	39.99 (7.88)	− 7.044	− 11.93 to − 2.159	0.006**	1.10
T1 vs. T3		42.01 (7.85)	− 9.058	− 16.44 to − 1.674	0.017*	1.41
*STS*						
T1 vs. T2	15.14 (6.44)	17.76 (6.27)	− 2.622	− 5.063 to − 0.181	0.032*	0.41
T1 vs. T3		19.53 (6.93)	− 4.387	− 7.402 to − 1.371	0.003**	0.68
T1 vs. T4		17.59 (8.52)	− 2.445	− 6.311 to 1.420	0.376	0.38
T1 vs. T5		19.07 (8.03)	− 3.924	− 7.607 to − 0.241	0.035*	0.61
*HGS-D*						
T1 vs. T2	29.07 (9.79)	32.82 (8.51)	− 0.146	− 0.263 to − 0.029	0.011*	0.38
T1 vs. T3		33.5 (8.69)	− 0.162	− 0.283 to − 0.040	0.007**	0.45
T1 vs. T4		31.35 (9.21)	− 0.088	− 0.232 to 0.057	0.429	0.23
T1 vs T5		35.09 (9.83)	− 0.207	− 0.338 to − 0.076	0.002**	0.62
*HGS-N*						
T1 vs. T2	25.43 (9.91)	30.85 (8.35)	− 5.424	− 8.985 to − 1.864	0.002**	0.55
T1 vs. T3		27.81 (8.41)	− 2.377	− 5.412 to 0.6578	0.172	0.24
T1 vs. T4		28.35 (8.27)	− 2.924	− 6.937 to 1.089	0.228	0.29
T1 vs. T5		31.59 (9.89)	− 6.16	− 10.47 to − 1.850	0.004**	0.62
*FACT-BMT*						
T1 vs. T2	116.4 (14.16)	117.7 (17.0)	− 1.216	− 7.092 to 4.659	> 0.99	0.09
T1 vs. T3		123.4 (15.12)	− 6.922	− 12.36 to − 1.481	0.01*	0.49
T1 vs. T4		121.5 (15.78)	− 5.008	− 14.26 to 4.249	0.583	0.36
T1 vs. T5		122.5 (17.83)	− 6.018	− 14.30 to 2.266	0.236	0.43
*FACT-G*						
T1 vs. T2	85.98 (10.98)	88.25 (12.52)	− 2.268	− 7.011 to 2.474	0.804	0.21
T1 vs. T3		91.23 (11.76)	− 5.245	− 9.130 to − 1.360	0.001**	0.48
T1 vs. T4		90.92 (10.91)	− 4.932	− 12.20 to 2.334	0.293	0.45
T1 vs. T5		91.02 (12.57)	− 5.033	− 11.64 to 1.578	0.195	0.46

*p < 0.05, **p < 0.01

Lower-body strength assessment on the Sit- to-stand (STS) Test was significantly higher immediately post-intervention (T2), and at T3 (M = 19.53, SD = 6.93, ES = 0.68) and T5 (M = 19.07, SD = 8.03, ES = 0.61) compared to T1 (M = 15.14, SD = 6.44; Fig. 2c). For the upper limb assessment, dominant hand grip was significantly stronger immediately post-intervention (T2), and at T3, and T5 (M = 35.09, SD = 9.83, ES = 0.62) than at T1 (M = 29.07, SD = 9.79; Fig. 2d). Non-dominant hand grip strength was also significantly stronger immediately post-intervention (T2), and T5 (M = 31.59, SD = 9.89, ES = 0.62) compared to T1 (M = 25.43, SD = 9.91; Fig. 2e).

A significantly higher self-reported QOL represented by the FACT-BMT total score was found at T3 (M = 123.37, SD = 15.12, ES = 0.49), when compared to T1 (M = 116.44, SD = 14.16; Fig. 3a). Similarly, FACT-G subscale was significantly improved at T3 (M = 91.23, SD = 11.76, ES = 0.48), when compared to T1 (M = 88.25, SD = 12.52; Fig. 3b) but not for FACT-BMT TOI subscale (data not shown).

There were no statistically significant differences on the Godin-Shepard Leisure Time Questionnaire, PSQI, and HADS (Additional file 4: Figs. S1–S4).

Eighty-four percent, 89% and 89% of the participants rated the video-conferencing platform used to deliver the intervention (Q5), the quality of the intervention (Q6), and their own participation (Q7) as very good or excellent (mean score > 4). MBSM and PE components were found to be very useful or higher by 57.9% and 78.9% of participants, respectively. Participants reported that their MBSM and PE habits had changed markedly or very markedly because of the intervention at 6 months post-baseline in 47%. (Additional file 4: Fig. S5). No adverse events were reported by participants during the duration of the pilot study.

## Discussion

We evaluated a 6-week personalised and supervised PE and MBSM telehealth program delivered to long term post HSCT patients with 12-month follow-up. Significant improvements in cardiovascular fitness, upper and lower body strength, and self-reported QOL (FACT-BMT) measures were noted at 3- and 12-months post-intervention. QOL indices indicated overall improvement in general quality of life (FACT-G) comprised of physical, emotional, social and functional wellbeing and BMT-related QOL (FACT-BMT); a reduction in transplant-related concerns. We did not find significant changes on leisure time activity usage, or measures of sleep quality. We also found this combined modality telehealth program with internet-based assessments was feasible, safe, and acceptable. About half of participants indicated at 6 months post-intervention that the program changed their self-reported habits for stress management and exercise for the better. Our findings demonstrating long term responses to the virtual intervention add to the existing literature of combined modality training programs in patients with cancer and post-HSCT [23, 25, 31, 32, 46]. To our knowledge, this study has the longest follow-up observation period for internet based combined modality training program reported to date. Findings suggest that such interventions may have long lasting health benefits.

This program offers an accessible, well-accepted, and potentially effective internet-based intervention to meet the needs of transplant survivors and is of interest as the availability of rehabilitation programs for cancer survivors remains suboptimal [47] The lack of healthcare accessibility and low adherence to recommended lifestyle interventions contributes significantly to poorer health outcomes. It is particularly relevant for many countries in which a large proportion of the population lives in rural and regional locations far from treatment centres. It also has the possibility to be adapted with relative ease to address the needs of minority non-English speaking groups who are known to have poorer outcomes than English-speakers in countries such as Australia.

Telehealth has been of particular relevance during the current COVID-19 pandemic, where challenges that were unique to vulnerable patient groups with low immunity are experienced more universally. Internet-based ways of working, socializing and receiving healthcare have been accelerated and accessed more widely in Australia and worldwide. Outcome findings of the efficacy and acceptability of health interventions delivered via telehealth are much needed and can facilitate goals to make rehabilitation programs accessible for more cancer survivors as part of an organized strategy [48].

The benefits of the program on measures of quality of life showed less longevity than improvements on the physical outcomes, and measured symptoms of psychological distress did not significantly improve at all. This may be as the intervention targets how the person manages the effects of these symptoms on their overall functioning and QOL, rather than directly targeting the symptoms themselves. We propose that future studies using MBSR should specifically measure symptoms of stress. Given participants with high initial scores on screening measures for anxiety and depression symptoms on the HADS were excluded and referred to treatment, it is also possible that a flooring effect was observed on this measure.

Our study has a number of limitations. This is a single arm supervised pilot study, and it is not possible to conclude whether the combined modality is required for efficacy in supervised programs. We cannot exclude motivation bias as patients with better telehealth familiarity or enthusiasm for exercise may have been more likely to consent to the study. This could affect the generalisability of the findings. Additionally, some improvements observed to 3 months post intervention and the benefits were not observed at 6 months. This leads us to hypothesize that the addition of motivation sessions between 3 and 6 months may lead to long term improvements across a wider range of outcomes, as suggested in a previous study by Vallerand et al. [49] We report findings of a single centre study with limited participants, and a multi-centre RCT is underway to address these issues and to further explore outcomes.

A practical consideration for future research is the significant variation in patients in this cohort in terms of recovery timeline and functioning. We found that patients who had reengaged in the workforce or moved away were less likely to participate and those with severe medical problems were ineligible. Some of our patients responded that they wished they had access to a program like this earlier in their care. A wide-ranging and flexible approach to clinical care is required to support longer term HSC transplant recovery and combined exercise and physiological interventions should be adaptive to patients’ changing needs.

## Conclusions

We demonstrated the feasibility and acceptance of a 6-week personalised combined modal telehealth program to allogeneic-HSCT survivors. The results of objective and subjective assessments demonstrated that improvements in physical and cardiovascular fitness, and self-reported quality of life were sustained to at least 3 months, and for some measures up to 12 months post-training, the longest follow-up observation period reported so far. The significance of these findings is particularly relevant today, where telemedicine is assuming a greater role than ever in delivering safe and effective healthcare to vulnerable populations such as meeting the challenges of the current pandemic. Implementation of such interventions awaits the confirmation of multi-centre randomised control trials.

## Supplementary Information

**Additional file 1**: Additional trial details including orientation session, exercise and MBSM interventions, outcome assessments and hand strength dynamometer calibration.

**Additional file 2**: Completed TIDier checklist with details of study interventions.

**Additional file 3**: Completed CERT Checklist with details of exercise intervention.

**Additional file 4**: **Fig. S1**. Participant scores on the Godin-Shepard Leisure Time Questionnaire, where higher scores indicate greated self-reported use of leisure time. **Fig. S2**. Participant scores on the Pittsburgh Sleep Quality Index (PQSI) where lower scores indicate improvements in sleep quality. **Fig. S3**. Participant scores on the Hospital Anxiety and Depression Scale (HADS) anxiety subscale, where reduced scores indicate improvements in self-reported symptoms of anxiety.Supplementary **Fig. S4**. Participant scores on the Hospital Anxiety and Depression Scale (HADS) depression subscale, where reduced scores indicate improvements in self-reported symptoms of depression. **Fig. S5**. Six-point Likert scale from questionnaire evaluating patient feedback at 6-months post training. Q1- Was the MBSM useful? Q2- Was the exercise component useful? Q3- Did MBSM change your practice? Q4- Did exercise change your habits? Q5- How do you rate telehealth (video) as a method of service provision? Q6- How would you rate the quality of service if it was a part of your treatment? Q7- Do you feel that your participation has been worthwhile?

## Data Availability

Requests for study data can be addressed to the corresponding author.
