# Supplementary material for: Potential benefits of a virtual, home-based combined exercise and mindfulness training program for HSC transplant survivors: a single-arm pilot study
Source: BMC Sports Sci Med Rehabil. 2022 Sep 5;14:167. doi: 10.1186/s13102-022-00554-7 (PMC9444110; doi:10.1186/s13102-022-00554-7)
Supplement: Supplementary file 1 — Additional file 1: Additional trial details including orientation session, exercise and MBSM interventions, outcome assessments and hand strength dynamometer calibration. [file 13102_2022_554_MOESM1_ESM.docx]

**Suppl A: Description of In-person Orientation Session**

**Individual Exercise Session**

- All participants completed the baseline outcome measures during this session. These measures were weight(kg), waist circumference(cm), 30s Sit to Stand test (number of repetitions), Bilateral grip strength(kg/F), and 6-minute walk test (distance(m) & Borg (RPE). Each participant’s results were recorded by the Exercise Physiologist and the participant was also educated on how to set up and perform each test from their home-base. Exercise history and the participants overall exercise goals were also discussed.

**Individual Psychological Session**

- All participants completed a semi-structured interview exploring their mental health history, current emotional state (mood/anxiety), current stressors, past and present psychological treatment (if any), existing stress management strategies, and social support networks.

**Questionnaire Assessment**

- All participants completed their questionnaire assessment (as described in Supplement D) during their in-person orientation session, in their individual meetings with the assessors. Each assessor asked the questions and recorded the answers electronically or on physical copies of the standardized questionnaires. Participants were able to ask for clarification from the assessor, and the assessor was able to provide feedback to the participant in vivo.

**Suppl B: Description of 6-week Telehealth Exercise Session**

- In the first session, the participant’s individually prescribed exercise program, based on the assessment at the orientation session, was demonstrated to them and resistance bands were provided. The participant attempted each home exercise with supervision via Skype and any issues or details were discussed at the time. If required, an exercise was modified to suit the participant’s abilities or needs. At the end of each session, the exercise goals for the week were discussed and set. All sessions lasted between 30-60 minutes
- The aerobic exercise component was individualized for each participant and could include walking, running, swimming or cycling for a duration of 20-30 min. The resistance exercise component included resistance bands and body-weight strength exercises for the upper and lower body, aiming for 2-3 sets of 8-12 repetitions. For these programs, the aim is to achieve an intensity level of RPE between 11 – 14 and perform 3 to 5 times per week.
- The subsequent sessions varied based on their feedback including the RPE scale and set goals (target: - moderate to high intensity level (RPE 11-14)). The barriers and issues such as difficulty, soreness or fatigue changes to the exercise program were demonstrated and/or attempted by the participant under supervision through Skype.
- Planning and problem-solving strategies were utilized to help to overcome barriers and issues such as difficulty, soreness or fatigue. Goals were set around these matters to be met for the following week. Any participant who had any medical issues arise during the program, had their program modified accordingly and medical advice was sought.
- At the last training sessions, there was a discussion as to how to progress or modify the exercise program over the following unsupervised period. Progression or modification methods discussed were similar to those employed during the supervised period. Participants were given education on safe methods to gradually increase the program, and not have an RPE rating greater than 17 for a prolonged period, which was defined individually dependent on their abilities. The potential contra-indicators to exercise were outlined in the handbook and explained to the participant. All participants were asked to report any pain, injuries or any other problems that impaired their ability to perform any activities for the remaining trial period.

**Suppl C: Description of 6-week Telehealth Mindfulness based Stress Management Sessions**

- Stress management training was conducted over six sessions delivered weekly via Skype. This training was adapted from Mindfulness Based Stress Reduction (MBSR)^1,2^ for delivery in a one-to-one setting.
- The first five sessions adhered to the following format: review of previous material, introduction to and practice of a mindfulness exercise, practice reflection, and time for questions. Each mindfulness exercise was practiced during the session using a standard script read aloud by the psychologist, with a new mindfulness exercise introduced per week. All sessions lasted between 30-60 minutes.
- Similar to MBSR and its adaptions for cancer populations^1, 2, 3^, participants in our intervention were introduced to a combination of formal and informal mindfulness exercises. Specifically, participants were introduced to five mindfulness exercises over the course of the training: mindfulness of breathing (focus on the breath), body scan (focus on body sensations), mindfulness of sounds (focus on environmental sounds), and mountain and lake meditations (focus on mountain imagery). All exercises were between 5 and 10 minutes long. One week of training was devoted to teaching participants to use mindfulness during daily activities such as eating or walking, and to use mindfulness-based “grounding” strategies during stressful situations.
- To facilitate practice at home, participants were emailed a mp3 recording of each mindfulness exercise. The recordings were identical to the scripts used during the session. Participants were instructed to practice daily, and self-reported adherence was recorded in their file each week.
- The final (6^th^) week of training included a review of all mindfulness exercises and goal setting for their mindfulness practice over the 3-month unsupervised period. Participants could choose a preferred exercise to continue practicing or could rotate between all exercises. Barriers to practice and strategies for overcoming these barriers were also identified
- For participants experiencing higher levels of stress identified prior or during training sessions, additional stress management techniques from the handbook were discussed and added to the mindfulness practice. Participants who developed more severe symptoms over the course of the trial were advised to discuss these issues with their regular psychologist, or to seek a referral to a psychologist per our institutional standard of care guideline requirements.

References:

1. Kabat-Zinn J. Full Catastrophe Living: Using the Wisdom of Your Body and Mind to Face Stress, Pain, and Illness. New York, NY: Delacourt; 1990.

2. Schell LK, Monsef I, Wöckel A, Skoetz N. Mindfulness-based stress reduction for women diagnosed with breast cancer. *Cochrane Database of Systematic Reviews* 2019, Issue 3. Art. No.: CD011518. DOI: 10.1002/14651858.CD011518.pub2.

3. Lengacher C, Reich R, Paterson C, Ramesar S, Park J, Alinat C et al. Examination of Broad Symptom Improvement Resulting From Mindfulness-Based Stress Reduction in Breast Cancer Survivors: A Randomized Controlled Trial. *Journal of Clinical Oncology*. 2016;34(24):2827-2834.

**Suppl D:- Description of assessments via videoconferencing and online self-assessments**

For 6-MWT^1^, participants were instructed to pre-measure a point-to-point lap on a flat surface at their home and use the same lap for each assessment. The trial co-ordinator kept the time for the test and recorded the number of laps completed and, the start and finish RPE via video link.

Hand Grip Strength (HGS) was measured with the provided dynamometer. Instructions and demonstrations were given to the participants according to the standard recommendations^2^. The participant was instructed to hold the dynamometer in their hand and squeeze with maximal force for one exhalation. The grip strength test consisted of three maximal voluntary contractions in each hand, with the force output in kilograms recorded, with the best taken for analysis.

The Sit-to-stand (STS) test^3^ was performed using the participant’s chair (same chair for all assessments), where they were instructed to begin in a seated position. The participant was asked to stand up fully and sit down (touch the chair) without using upper body assistance on the chair for 30 seconds. The number of times the participant stood up fully was counted and recorded by the trial coordinator.

Other secondary outcomes included weight, waist circumference and participant self-report of physical activity, quality of life, and functional and psychological wellbeing via online questionnaires. These questionnaires included (in order of presentation to participants): Hospital Anxiety and Depression Score (HADS)^4^, Depression, Anxiety and Stress Scale (DASS-21)^5^, Functional Assessment of Cancer Therapy- Bone Marrow Transplant (FACT-BMT)^6^, Pittsburgh Sleep Quality Index (PSQI)^8^, and Godin-Shephard Leisure Time Physical Activity Questionnaire (GS-LTPAQ)^9^.

Initial testing demonstrated that the online questionnaires could be completed within 15-20 minutes.

References:

1. Enright PL. *The six-minute walk test*. Respir Care. 2003 Aug;48(8):783-5. PMID: 12890299.

2. American Society of Hand Therapists. *Clinical assessment recommendations.* Chicago: The Society; 1992

3. Alcazar J, Losa-Reyna J, Rodriguez-Lopez C, Alfaro-Acha A, Rodriguez-Mañas L, Ara I, García-García FJ, Alegre LM. The sit-to-stand muscle power test: An easy, inexpensive and portable procedure to assess muscle power in older people. Exp Gerontol. 2018 Oct 2;112:38-43. doi: 10.1016/j.exger.2018.08.006.

4. Zigmond AS, Snaith RP. The hospital anxiety and depression scale. *Acta Psychiatr Scand*. 1983;67(6):361-370.

5. Lovibond PF, Lovibond SH. The structure of negative emotional states: Comparison of the Depression Anxiety Stress Scales (DASS) with the Beck Depression and Anxiety Inventories. *Behav Res Ther*. 1995;33(3):335-343. doi:https://doi.org/10.1016/0005-7967(94)00075-U.

6. McQuellon RP, Russell GB, Cella DF, et al. Quality of life measurement in bone marrow transplantation: development of the Functional Assessment of Cancer Therapy-Bone Marrow Transplant (FACT-BMT) scale. *Bone Marrow Transplant*. 1997;19(4):357.

7. Yellen SB, Cella DF, Webster K, Blendowski C, Kaplan E. Measuring fatigue and other anemia-related symptoms with the Functional Assessment of Cancer Therapy (FACT) measurement system. *J Pain Symptom Manage*. 1997;13(2):63-74.

8. Buysse DJ, Reynolds III CF, Monk TH, Berman SR, Kupfer DJ. The Pittsburgh Sleep Quality Index: a new instrument for psychiatric practice and research. *Psychiatry Res*. 1989;28(2):193-213.

9. Godin G, Shephard RJ. A simple method to assess exercise behavior in the community. *Can J Appl Sport Sci*. 1985;10(3):141-146.

**Suppl E:- Description of grip strength dynamometer calibration**

To ensure reliable and accurate grip strength results all grip strength dynamometers were calibrated prior to being used and given to participants. The grip strength dynamometer was calibrated using a standard weight method (1), repeated three times, utilizing 2 researchers to validate the result. The weights used were 1.5kg and 5kg weight. The grip strength dynamometer had to return a value within 0.2kg for each of the standard weights in all 3 tests to pass calibration. If the grip strength dynamometer did not meet these standards for calibration, the dynamometer was returned to the manufacturer.

Participants were instructed to keep the grip strength dynamometer in the box and was only to be used for trial assessment purposes.

1. Ewing-Fess, E., "A Method for Checking Jamar Dynamometer Calibration," Journal of Hand Therapy 1.1:28-32, 1987.
