# Supplementary material for: Potential benefits of a virtual, home-based combined exercise and mindfulness training program for HSC transplant survivors: a single-arm pilot study
Source: BMC Sports Sci Med Rehabil. 2022 Sep 5;14:167. doi: 10.1186/s13102-022-00554-7 (PMC9444110; doi:10.1186/s13102-022-00554-7)
Supplement: Supplementary file 3 — Additional file 3: Completed CERT Checklist with details of exercise intervention. [file 13102_2022_554_MOESM3_ESM.docx]

CERT ✓***C***onsensus on ***E***xercise ***R***eporting ***T***emplate

A Checklist for what to include when reporting exercise programs

| **Section/Topic** | **Item #** | **Checklist item** | **Location** ** | |
| --- | --- | --- | --- | --- |
|  |  |  | Primary paper (page, table,  appendix) | † Other (paper or protocol, website  (URL) |
| **WHAT: materials** | 1 | Detailed description of the type of exercise equipment (e.g. weights, exercise equipment supp A pg1  such as machines, treadmill, bicycle ergometer etc) | |  |
| **WHO: provider** | 2 | Detailed description of the qualifications, teaching/supervising expertise, and/or training  Methods pg3  undertaken by the exercise instructor  Supp B pg1 | |  |
| **HOW: delivery** | 3 | Describe whether exercises are performed individually or in a group |  |  |
|  | 4 | Describe whether exercises are supervised or unsupervised and how they are delivered  Supp B pg1 | |  |
|  | 5 | Detailed description of how adherence to exercise is measured and reported | Methods pg5 |  |
|  | 6 | Detailed description of motivation strategies | Supp B pg1 |  |
|  | 7a | Detailed description of the decision rule(s) for determining exercise progression  Supp B pg1-2 | | Supp B pg1-2 |
|  | 7b | Detailed description of how the exercise program was progressed |  | *no illustrations,available Details were included in the hard copy handbook given to participants. |
|  | 8 | Detailed description of each exercise to enable replication (e.g. photographs, illustrations ,  video etc) | | Methods pg4 |
|  | 9 | Detailed description of any home program component (e.g. other exercises, stretching etc) | | Methods pg4 |
|  | 10 | Describe whether there are any non-exercise components (e.g. education, cognitive  behavioural therapy, massage etc) | |  |
|  | 11 | Describe the type and number of adverse events that occurred during exercise | Discussion pg9 |  |

Methods pg4 - virtual

| **WHERE: location** | 12 | Describe the setting in which the exercises are performed  Methods pg4 |
| --- | --- | --- |
| **WHEN, HOW**  **MUCH: dosage** | 13 | Detailed description of the exercise intervention including, but not limited to, number of  exercise repetitions/sets/sessions, session duration, intervention/program duration etc |
| **TAILORING:**  **what, how** | 14a | Describe whether the exercises are generic (one size fits all) or tailored whether tailored to the individual  Methods pg4  Methods pg4 - tailored |
|  | 14b | Detailed description of how exercises are tailored to the individual |
|  | 15 | Describe the decision rule for determining the starting level at which people commence an exercise program (such as beginner, intermediate, advanced etc)  Methods pg4 – skill level, exercise history |
| **HOW WELL:**  **planned, actual** | 16a | Describe how adherence or fidelity to the exercise intervention is assessed/measured  Discussion pg8  Methods pg5 |
|  | 16b | Describe the extent to which the intervention was delivered as planned |

***It is recommended that this checklist is used in conjunction with the Explanation and Elaboration Statement which is a guide each item in the CERT Checklist**

The CERT Checklist is designed for reporting details of an exercise intervention. The CERT Checklist should be used in conjunction with a reporting checklist appropriate for the study type e.g. the CONSORT Statement (www.consort‐statement.org) for randomised controlled trials, the SPIRIT Statement (www.spirit‐statement.org) for a clinical trial protocol. For further guidance regarding reporting guidelines please consult the EQUATOR network (www.equator‐network.org)

****** Authors – please use N/A if an item is not applicable Reviewers – please use ”?” if information is not provided or not/insufficiently reported

**^†^** If the information is not provided in the primary paper that is under consideration, please provide details of where this information is available e.g. in a published protocol, published papers (provide citation details) or on a website (provide the URL).
