# Supplementary figures and images for: Potential benefits of a virtual, home-based combined exercise and mindfulness training program for HSC transplant survivors: a single-arm pilot study
Source: BMC Sports Sci Med Rehabil. 2022 Sep 5;14:167. doi: 10.1186/s13102-022-00554-7 (PMC9444110; doi:10.1186/s13102-022-00554-7)

Supplementary

Fig 1

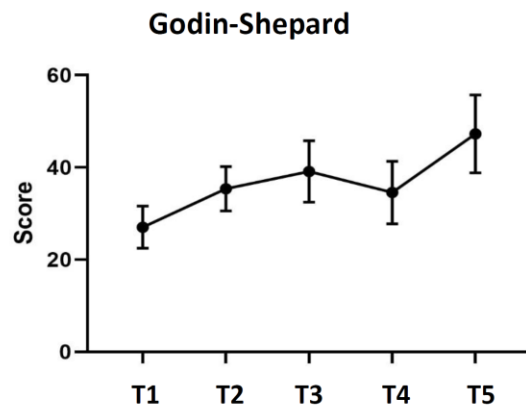

Fig 2

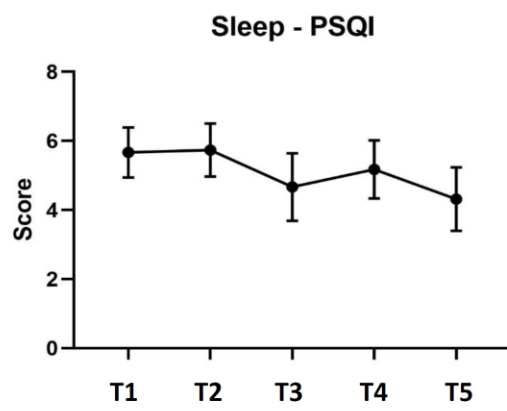

Fig 3

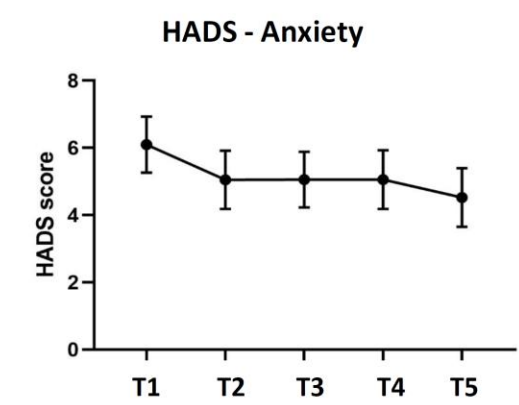

Fig 4

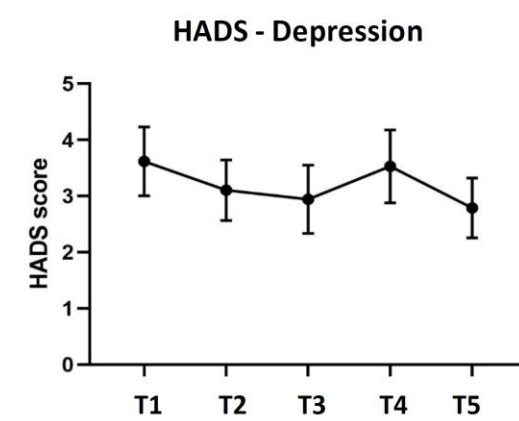

Fig 5

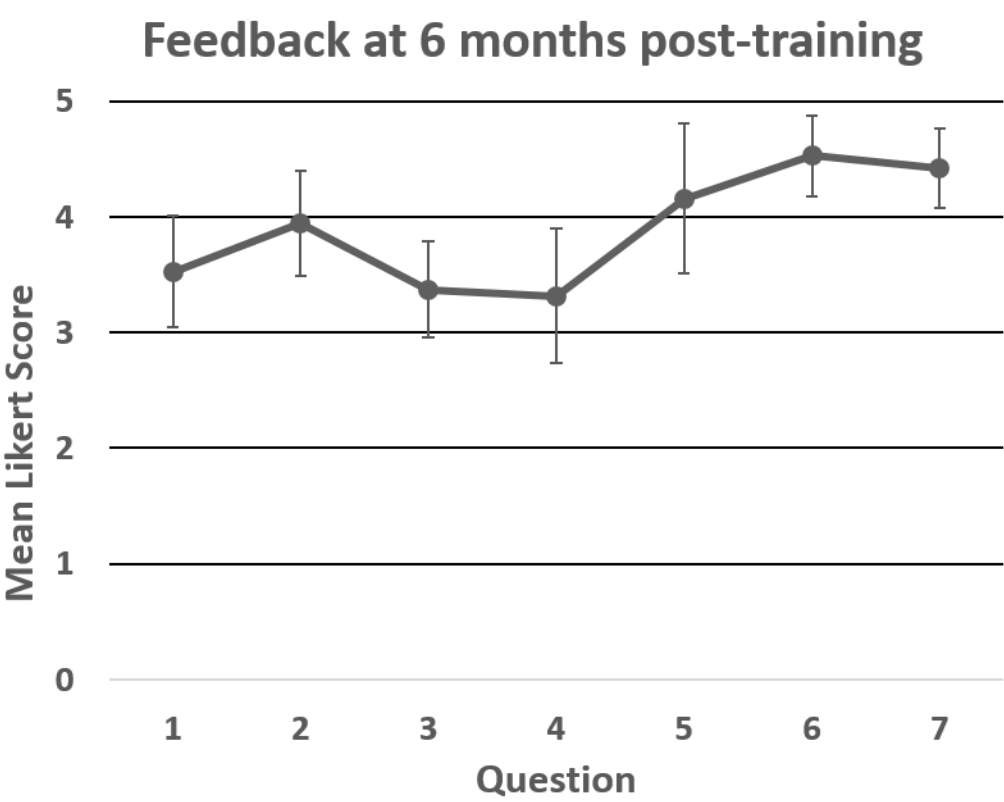

Supplement: Supplementary file 4 — Additional file 4: Fig. S1. Participant scores on the Godin-Shepard Leisure Time Questionnaire, where higher scores indicate greated self-reported use of leisure time. Fig. S2. Participant scores on the Pittsburgh Sleep Quality Index (PQSI) where lower scores indicate improvements in sleep quality. Fig. S3. Participant scores on the Hospital Anxiety and Depression Scale (HADS) anxiety subscale, where reduced scores indicate improvements in self-reported symptoms of anxiety.Supplementary Fig. S4. Participant scores on the Hospital Anxiety and Depression Scale (HADS) depression subscale, where reduced scores indicate improvements in self-reported symptoms of depression. Fig. S5. Six-point Likert scale from questionnaire evaluating patient feedback at 6-months post training. Q1- Was the MBSM useful? Q2- Was the exercise component useful? Q3- Did MBSM change your practice? Q4- Did exercise change your habits? Q5- How do you rate telehealth (video) as a method of service provision? Q6- How would you rate the quality of service if it was a part of your treatment? Q7- Do you feel that your participation has been worthwhile? [file 13102_2022_554_MOESM4_ESM.pdf]
